# Challenges, consequences, and lessons for way–outs to emergencies at hospitals: a systematic review study

**DOI:** 10.1186/s12873-019-0275-9

**Published:** 2019-10-30

**Authors:** Hamid Reza Rasouli, Ali Aliakbar Esfahani, Mohsen Abbasi Farajzadeh

**Affiliations:** 1 Trauma Research Center, Baqiyatallah University of Medical Sciences, Tehran, Iran; 2 Marine Medicine Research Center, Baqiyatallah University of Medical Sciences, Tehran, Iran

**Keywords:** Emergency department, Emergency crowding, Overcrowding

## Abstract

**Background:**

Emergency Department (ED) overcrowding adversely affects patients’ health, accessibility, and quality of healthcare systems for communities. Several studies have addressed this issue. This study aimed to conduct a systematic review study concerning challenges, lessons and way outs of clinical emergencies at hospitals.

**Methods:**

Original research articles on crowding of emergencies at hospitals published from 1st January 2007, and 1st August 2018 were utilized. Relevant studies from the PubMed and EMBASE databases were assessed using suitable keywords. Two reviewers independently screened the titles, abstracts and the methodological validity of the records using data extraction format before their inclusion in the final review. Discussions with the senior faculty member were used to resolve any disagreements among the reviewers during the assessment phase.

**Results:**

Out of the total 117 articles in the final record, we excluded 11 of them because of poor quality. Thus, this systematic review synthesized the reports of 106 original articles. Overall 14, 55 and 29 of the reviewed refer to causes, effects, and solutions of ED crowding, respectively. The review also included four articles on both causes and effects and another four on causes and solutions. Multiple individual patients and healthcare system related challenges, experiences and responses to crowding and its consequences are comprehensively synthesized.

**Conclusion:**

ED overcrowding is a multi-facet issue which affects by patient-related factors and emergency service delivery. Crowding of the EDs adversely affected individual patients, healthcare delivery systems and communities. The identified issues concern organizational managers, leadership, and operational level actions to reduce crowding and improve emergency healthcare outcomes efficiently.

## Introduction

The requirement of emergency healthcare service for people is an ongoing problem [1–5]. The ED must provide emergency care to the large populations. Nevertheless, the safe-networking of the emergency care added to the complexity of the role because the ED should provide services to the users regardless of their insurance and socioeconomic status [1, 2]. Furthermore, the ED might be the only reference of health care services to people, particularly in rural areas [1, 2].

Studies demonstrated increasing emergency healthcare services use due to the raised accidental injuries. Nonetheless, the potentials for emergency healthcare systems have not been completely developed [3–5]. This situation generates the crowding and overcrowding of the EDs which in turn posed a reduction of quality healthcare services and results. Thus, crowding is a condition when demand for emergency healthcare services exceeds from the available resources [3, 6].

Overcrowding of the emergency healthcare services has adverse results to the patients, the healthcare services and the population [7, 8]. Delay in healthcare services provided to patients can hurt the quality of the emergency services and also their outcomes [9]. Overcrowding of the ED could also generate the adverse consequences of standards services preparation, which in turn might outcomes in patients way out the EDs without obtaining the needed aids [7–9]. Despite the contributions to the understanding of medical emergencies [10, 11]. This systematic review aimed to assess the causes and challenges of ED crowding, the experiences of emergency patients, emergency care providers, and healthcare systems, and the solutions to ED crowding and their consequences since 2007 globally. The outcomes are expected to contribute inputs to decision-makers to contextualize practical solutions to promote the quality of medical emergency services and to the scientific readership.

## Materials and methods

### Search strategy

In this study, the definition for “crowding” from the American College of Emergency Physicians which states “Crowding occurs when the identified need for emergency services exceeds available resources for patient care in the emergency department, hospital, or both” was used. Then, articles related to crowding in EDs published in English between January 1, 2007, and August 1, 2018, from the MEDLINE through PubMed and Embase electronic databases were searched. Search keywords and phrases utilized were: ‘emergency’, ‘emergency medicine’, ‘pediatric emergency medicine’, ‘emergency medical services’, ‘emergency room’, ‘hospital emergency services’, ‘emergency health services’, ‘emergency department’, ‘emergency ward’, ‘EW’, ‘ED’, “AND”, ‘crowding’, ‘overcrowded’, ‘crowded’ ‘overcrowding’, ‘divert’, ‘diversion’, ‘congestion’, ‘surged’, ‘surging’, ‘capacity’, ‘crises’, ‘crisis’, ‘occupancy’, ‘hospital bed utilization’, ‘bed’, ‘utilization’, “OR”, “AND”, ‘effects’, ‘consequences’, ‘outcomes’,, affects’, ‘harm’, ‘impact’, ‘mortality’, ‘challenges’, ‘causes’ ‘strategies’, ‘solutions’, ‘lessons’, ‘interventions’, ‘negative’.

### Data collection and quality assessment

**Fig. 1:**
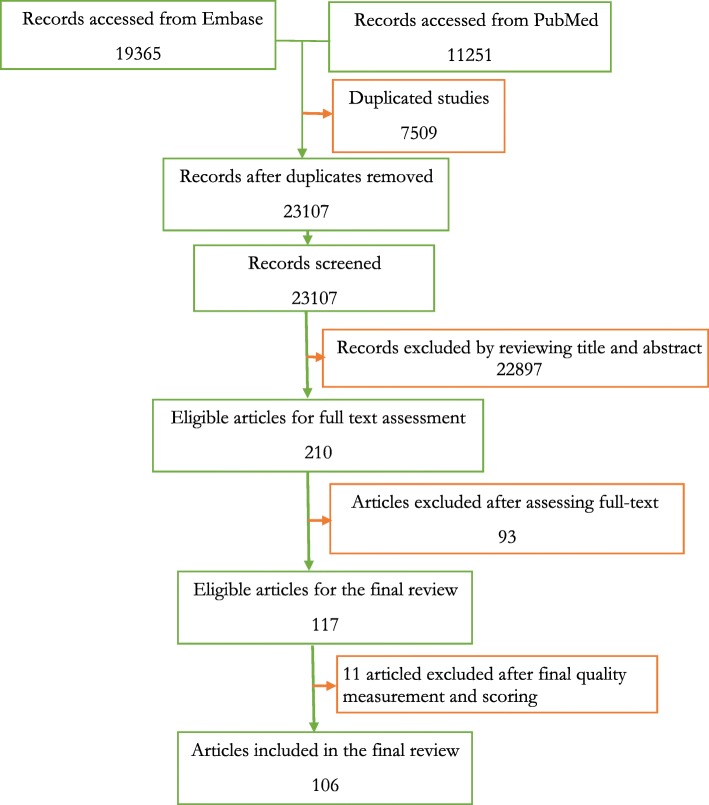
Study selection flowchart shows the database explorations, abstracts selected and the records included

Two reviewers (HRR AND MAF) independently screened the titles, abstracts and the methodological validity of the records using data extraction format before their inclusion in the final review. Discussions with the senior faculty member (AAE) were used to resolve any disagreements among the reviewers during the assessment phase. A total of 117 articles were eligible for the review (Fig. 1). The studies using the standardized Critical Appraisal Skills Programme (CASP) for the Cohort Studies [12], Qualitative Studies [13], and Systematic Reviews [14] were evaluated. Moreover, the Joanna Briggs Institute Meta-Analysis of Statistics Assessment and Review Instrument for studies which employed other designs was utilized [15]. Finally, 11 studies with an eligibility evaluation score of fewer than 0.33 points (< 33%), the final review was based on 106 studies.

The methodology part of the eligible articles was further assessed using the R ‘wordcloud’ software package to gain insight into the keywords in the abstracts [16]. We identified the focus of the studies and categorized the articles into those related to causes, effects, and solutions of crowding, and some form of their combinations.

## Results

**Fig. 2:**
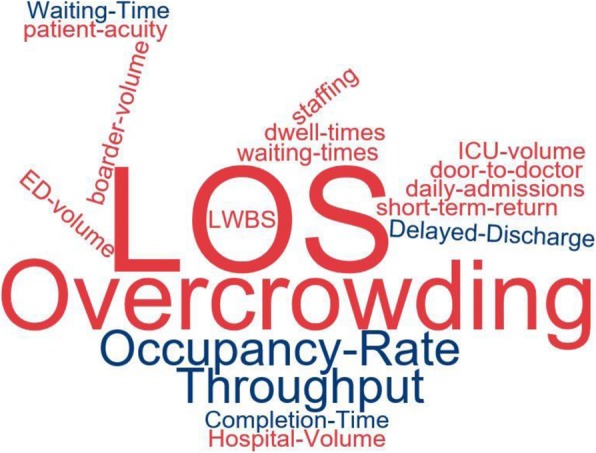
R ‘wordcloud’ of some of keywords in abstracts of the articles eligible for final review and their frequencies

Out of the total 106 eligible peer-reviewed original articles included in the final review, 14, 55, and 29 of them were on causes, effects, and solution of/to crowding of the ED, respectively. The articles reported on causes and effects were four [3, 17–19], and the remaining were on causes and solutions to crowding [9, 20–22] of/to crowding. Further assessment of the abstracts of the final articles using the R ‘wordcloud’ software package indicated some of the keywords and their frequencies (Fig. 2).

### Causes of EDs crowding

Studies reported different factors affecting the EDs crowding. Table 1 presented the individual patients, inputs (both human and material) and the service delivery processes related factors that influenced crowding either directly or indirectly. Male gender, being young (< 18 years) [18], and old age [23, 24, 35] were a patient-related factor associated with EDs crowding. The lifestyle of the emergency patient such as alcoholism also led a frequent hospital visit and crowding [25].

**Table 1:** Leading causes of EDs crowding

Patient-related factors [18, 20, 23–25]	
• Being a critically ill	
• Age (being child and aging)	
• Male gender	
• Lifestyle such alcoholism	
Emergency service delivery related causes	
a) *Emergency patient flow and emergency care related* [3, 8, 17, 19, 22, 26–30]	
• Delay in discharging admitted patients	
• Severe emergency condition	
• A high proportion of emergency patients	
• Long waiting of emergency patients for diagnostic test results	
b) *Emergency department staff related causes* [22, 25, 27, 28, 31–34]	
• Wrong diagnosis	
• Delay of consultants	
• Delay of staff to provide emergency services	
• Delay in transferring patients to inpatient ward	
• Shortage of emergency care providers	
c) *Premises and materials of ED-related factors* [9, 27]	
• Shortage of beds for admitting emergency patients	
Other hospital services delivery related causes [3, 19, 22, 27, 29, 31]	
• Delay in laboratory and imaging investigations	
• Delay in diagnostic test results/reports	
• The high number of patients in the waiting room of a hospital	
• The reluctance of hospital staff to admit patients from ED	

The high number of emergency patients and the seriousness of their conditions have hindered the timely admissions of the emergency patients and led to EDs overcrowding [3]. The increased waiting or boarding time of patients in the ED, the delay in discharging of the admitted patients [8, 26], and the high non-urgent patient flow to the hospitals [17, 19] were other identified factors that contributed to the EDs crowding. Furthermore, the delay in diagnostics and diagnostic test result reports [19, 24, 27, 31] and the delay in initiating treatment to emergency patients [32] were among the emergency service delivery related factors associated with the increased the length of patients waiting time that resulted in EDs crowding. The slow or delayed emergency patient admission process to the ED and inpatient wards [22, 33], and the prolonged laboratory and radiology testing influenced the crowding [22]. Shortage of emergency care staffs [22, 28] and hospital beds for admission of emergency patients [9, 27], and the reluctance of hospital staff to admit the emergency [29] were identified contributors to the EDs crowding.

### Effects of EDs crowding

The outcomes of overcrowding of ED are multilevel including effect on patients’ health consequences, healthcare delivery system and the communities. Table 2 shows the mainly related outcomes of the ED overcrowding. ED overcrowding caused delay treatment to patients and subsequently increased risks of not being precisely examined by the healthcare workers [19, 32, 36–40]. The patients through many walkouts may react to delay healthcare services and the overcrowding [41]. These situations could have an adverse effect of patients’ disease [50] and subsequently another consequences including re-admissions [51, 62], hospitalizations [50, 52, 53], healthcare costs [54], patients’ satisfaction [42–45], medical malpractices, morbidity [46–49], and patients’ mortality [50–61].

**Table 2:** Adverse consequences of EDs crowding

Adverse consequences on patients	
• Increase delay to treatment, patients LWBS [19, 32, 36–40] and subsequently walkouts due to perceived ED LOS [41] • Increase dissatisfaction [42–45] and medical malpractice [46–49] • Increase adverse effect [50] and deaths [50–61] • Increase readmissions [51, 62], hospitalization [50, 52, 53] and costs for healthcare [54]	
Adverse consequences on healthcare delivery system	
• Increase workload [63], delay service provision/decision making and increased ED LOS [53, 54, 56, 60, 61, 63–78] • Increase delay to management of outpatients [79] and overuse of ED facilities [64] • Decrease efficiency, and increase costs of healthcare [17, 36, 40, 50, 61, 80, 81] • Decrease consideration for infection prevention and control measures [82] • Decrease time and precise to examination patients’ conditions [71], compliance to standardization of healthcare [52] and quality of healthcare [39, 42, 63, 83–87] • Discharging of patients with high-risk clinical features [51] and diverting of patients to proper facilities [18] • Increase patients readmission rate [42] and admission rate to hospital wards [64] Decrease discharging rate of patients [37, 51, 57, 59, 66, 79, 88] and admission of patients [89]	

The discharging of patients especially with high-risk clinical characteristics [51] and misleading the patients to other departments [18] have adversely impacted the patients’ health consequences. These situations could increase the time of accepting and transferring the outpatients [79] and increase patient’s admission and re-admission rates [42, 61, 64, 89] consequently a reduced discharging rate of patients [51]. The increased hospitalization of patients caused overuse of all facilities [64].

The high workload caused prolonged service preparation and clinical decision making and raised patients’ LOS [53, 54, 56, 60, 61, 63–78]. These conditions have adversely affected the quality of services and performance [17, 36, 40, 50, 61, 80, 81, 83].

### Solutions to ED crowding

Medical emergencies and their negative consequences were of concern and several studies identified or suggested different approaches (strategies, and tactics), and solutions to reduces or prevent ED crowding and related consequences. The approaches can be broadly categorized into the organization or management level and operation level interventions (Table 3).

**Table 3:** Solutions of EDs crowding

Organization or management level solutions	
• Executive leadership involvement, hospital-wide coordinated strategies, data-driven management, and performance accountability [90]	
• Implementing emergency patient transfer network system (RTNS) [91]	
• Implementing Lean/Six Sigma Method [92]	
• Implementing an independent capacity protocol [93, 94]	
• Forecasting ED crowding [95]	
Operational level solutions	
a. Staffing and motivation	
• Pay for performance [96]	
• Staffing ED with qualified professionals [97, 98]	
b. Operational level strategies and tactics	
• Developing evidence-based admission criteria [21]	
• Implementing Electronic Blockage System (EBS) [99]	
• Implementing smoothing strategy [100]	
• Using capacity alert escalation calls [20]	
• Applying Discrete Event Simulation (DES) model [22]	
• Improving leadership of ED [101]	
• Implementing contingency strategy [102]	
• Using management-support multimodal hospital-wide interventions [103]	
• Implementing four-hour-rule for emergency care [104]	
• Introducing of Stat Lab [105]	
• Implementing Code Help Regulation [106]	
• Using a dashboard to provide real-time information about crowding [107]	
c. Service delivery process	
• Acute care emergency surgery service provision [108]	
• Whole week emergency service delivery [109]	
• Implementing triage by physicians [110]	
• Introducing efficient patient discharging process [111]	
• High-turnover utility bed management [112]	
• Implementing Timely Quality Care [113]	
• Implementing an improved ED patient flow [114]	
d. Other services	
• Enhanced primary care [21, 115]	
• Optimizing translation services [21]	
e. Premises	
• Expanding or opening additional EDs [116, 117]	
• Hallway emergency bed [9]	
• Increasing hospital bed capacity [118]	

#### Organization/management level solutions

Involving the executive leadership, implementing of hospital-wide coordinated strategies, strengthening evidence-based management and performance accountability [90] were suggested solutions to reduce the LOS of patients at EDs. Other approaches for reducing the ED crowding included implementing a coordinated patient transfer networking system (RTNS) [91] and an independent capacity protocol [93, 94]. Furthermore, the application of lean principles/Six Sigma in service delivery [92], and forecasting ED crowding [95] were strategies to shorten patient discharging and boarding hours.

#### Operational level solutions

Several studies recommended or identified interventions involving the clinical staff, operational level strategies and tactics, service delivery processes, healthcare infrastructure, and other services related factors that contributed or would contribute to the solution of ED crowding and its negative aftermaths.

- ***Staffing the ED and motivating the staffs:*** Introducing the pay for performance mechanism [96] and assigning ED residents [97] can contribute to the reduction of LOS of patients. Thus, the allocation of residents at the ED not only reduces the waiting time but also reduces the number of patients LWBS [98].
- ***Operational level strategies and tactics****:* Studies identified several operational-level means to reduce or prevent ED crowding. The methods included evidence-based patient admission [21], the application of a Discrete Event Simulation (DES) model [22], improving the emergency patient flow coordination leadership [101], introducing a Stat Lab within the ED [105], and implementing specific hospital-level action plans (Code Help Regulation) [106]. The use of Electronic Blockage System (EBS) (a form of triage system) contributed to the reduction of the ED crowding and the facilitation of patients admissions [99].

The application of management-support multimodal hospital-wide interventions decreases the ED occupancy and increase the four-hour performance without compromising the quality of care [103]. Following the four-hour-rule in deciding for admitting or discharging of patients also reduces patient death [104]. Furthermore, properly utilizing the unused capacity (smoothing strategy) [100], and using the capacity alert escalation calls [20] contribute to the reduction of the ED crowding by lowering ED bed occupancy rates. Assessing the patients in the waiting room was a feasible approach to reduce the ED crowding [102]. Besides, the use a dashboard provides real-time information which leads to actions towards preventing crowding [107].

c. ***Service delivery process***: Some interventions focused on emergency service delivery can reduce crowding. For example, initiating an acute care emergency surgery service, improving the ED patient flow and introducing an efficient patient discharging process reduce the ED bed occupancy and LOS [108, 111, 114]. The high-turnover utility bed management can also decrease ambulance diversion hours and LOS of patients [112]. The practice of triage by physicians reduce the patients LWBS in the EDs [110]. Other interventions which contribute to the reduction of crowding included whole week emergency service delivery [109] and implementing Timely Quality Care [113].
d. ***Other services:*** Improving other services such as enhancing primary care [21, 115], optimizing translation services concerning patients’ issues [21] and an engagement on specialists in the outpatient environment [116, 117] contribute to the reduction of ED LOS and crowding.
e. ***Premises:*** The high emergency patient flow forced the healthcare delivery system to address related issues. Expanding or opening additional EDs were suggested to reduce patients LWBS and boarding hours [116, 117]. Others followed hallway emergency bed policy [9] and increasing hospital bed capacity [118] to reduce waiting time and ED crowding.

## Discussion

Patients in the extreme the age categories (being a child and the elderly) [18, 23–25, 35], might have contributed to the crowding due to reduced physical mobility and the involvement of their relatives and the different emergency care expertise in their care. Among other personal factors, alcoholic patients who present to the ED [25] might also have limited mobility or might be unable to provide self-care. Being a male patient was also associated with frequent ED visits, while the exact relationship was not clear [18]. While a recent systematic review did not report concerning the relationship between Individual characteristics and crowding [119], our systematic review revealed an association between age and ED crowding. Nevertheless, being male, arriving of emergency patients during the weekends, and being adult non-trauma patients were reported to be linked with short LOS.

The seriousness of the emergency condition, the high flow of emergency patients, and the involvement of the different actors (relatives and health professionals) affect the service delivery process [3, 26–29, 120]. These conditions are more likely to cause a delay in emergency care provision [33], admitting and discharging of patients [26], and an increase in the waiting time of patients [120], in which all of them can lead to increased ED crowding. The high flow of in and out of the emergency patients including the inpatient boarding [10] and the presence of urgent and complex emergency conditions of the patients usually lead to crowding of the ED [120]. The prolonged LOS in EDs related to some causes [21] and the large volume of emergency patients flow to the ED are a common reason for crowding.

Several healthcare providers related factor including wrong diagnosis, delay in consultants to see emergency patients, delay in services provision and transferring of patients to inpatient wards, and a shortage of emergency healthcare providers [22, 25, 27, 28, 31–34] contributed to the ED crowding. Other previous systematic reviews also identified inadequate staffing including the shortage of emergency care nursing staff and delays in clinical decisions as causes of ED crowding [10, 11].

The inadequacy of beds at the ED for admitting patients was health infrastructure related causes of crowding [9, 27]. Similarly, other systematic reviews also identified the shortage of beds to be associated with ED crowding [10, 11]. The high flow of emergency patients to the ED who may require admissions and the limited number of beds in the ED leads to crowding and poor healthcare outcomes.

The delay in laboratory investigations, diagnostic imaging and in reporting diagnostic test results contribute to ED crowding [24, 27]. The shortage of materials and other resources can lead to delay in laboratory test results [31]. The high number of emergency patients [19], the reluctance of the hospital staff to admit emergency patients [29] and the inadequate number of emergency patient admissions [10, 11] were also reasons for crowding.

The results showed ED overcrowding related to delay treatment and increased risks not being seen for patients [19, 32, 36–40]. These conditions have an adverse effect of their disease [50], readmissions [51, 62], hospitalizations [50, 52, 53], healthcare costs [54], patients’ satisfaction [42–45]. In a systematic review by Morley et al. (2018) showed prolonged patient evaluation and preparation of care could adversely affect death rate, medical malpractice and patient satisfaction [11]. Also, in another systematic review study (2008) showed EDs overcrowding negatively affected patients’ mortality, quality of healthcare, and costs of services [10].

Our results demonstrated that ED overcrowding negatively effects on patients’ health, healthcare delivery services and the communities [42–49, 52–61, 64, 79, 89]. The high workload caused prolonged healthcare services and clinical decision making and raised patients’ LOS [53, 54, 56, 60, 61, 63–78]. Several emergency healthcare systems associated outcomes evaluated by Morley et al. study (2018) for chiefly they concentrated on inpatient LOS and ED LOS [11].

The involvement of the executive leaders, the use of hospital-wide coordinated approaches, and evidence-based management and performance accountability were some of the strategies implemented to reduce ED crowding and its consequences [90]. The use of a coordinated emergency patient transfer network system [91], and an independent capacity protocol [93, 94] were as strategies for reducing ED LOS of patients. Furthermore, the lean principles/Six Sigma Method in service delivery [92] and forecasting of ED crowding [95] believed to shorten patient discharging and boarding hours. Another systematic review study also identified leadership program/support and alternative admission policies as solutions to crowding [11].

Several specific technical (front-line) level tools or solutions have been identified. The staffing of the ED with qualified professionals [98] and the use of motivational mechanisms such as pay for performance [96] contributed to the alleviation of ED crowding. Developing and using evidence-based admission criteria [21], implementing Electronic Blockage System [99] and smoothing strategy [100], using capacity alert escalation calls [20] and applying Discrete Event Simulation (DES) model [22] were also the tactics applied to facilitate emergency service delivery and reduce crowding. Improving leadership of the ED [101], evaluating emergency patients in the waiting room as a contingency strategy [102], and using management-support multimodal hospital-wide interventions [103] were other reported tactics. Furthermore, implementing four-hour-rule for emergency care [104], introducing Stat Lab [105], implementing Code Help Regulation [106] and Using a dashboard to provide real-time information about crowding [107] were proposed operational tactics to the reduce ED crowding and related consequences.

Studies identified several technical level (or front-line) measures or solutions targeting the alleviation of ED crowding. Staffing the ED with qualified professionals [98] and pay for performance [96] were among others. Other tactics which aimed to to facilitate emergency service delivery and reduce crowding included: developing and using evidence-based admission criteria [21], implementing Electronic Blockage System [99], and smoothing strategy [100], using capacity alert escalation calls [20] and applying Discrete Event Simulation (DES) model [22]. Improving leadership of the ED [101], evaluating emergency patients in the waiting room as a contingency strategy [102], and using management-support multimodal hospital-wide interventions [103] were other reported tactics. Furthermore, implementing four-hour-rule for emergency care [104], introducing Stat Lab [105], implementing Code Help Regulation [106] and Using a dashboard to provide real-time information about crowding [107] were proposed operational level tactics to the reduce ED crowding and related consequences.

Operational interventions targeting the service delivery processes were also identified. Initiating an acute care emergency surgery service, improving ED patient flow and introducing an efficient patient discharging process could reduce the ED bed occupancy rate and LOS [108, 111, 114]. The high-turnover utility bed management [112], the practice of triage by physicians [110], whole week emergency service delivery [109], and implementing Timely Quality Care [113] contributed to the improvements in service delivery and reduction of crowding. A recent systematic review also identified different crowding measure, social interventions, fast track, ED nurse flow coordinator as operational level solutions to ED crowding [11].

Initiating an acute care emergency surgery service, improving ED patient flow, and introducing an efficient patient discharging process [108, 111, 114] were recommended interventions targeting improvement in service delivery processes and reduction in ED bed occupancy rate and LOS. High-turnover utility bed management [112], practicing triage by physicians [110], whole week emergency service delivery [109], and implementing Timely Quality Care [113] were also frontline interventions that contribute to improved service delivery and reduced crowding. A recent systematic review also identified different crowding measure, social interventions, fast track, and ED nurse flow coordinator as operational level solutions to ED crowding [11].

The emergency care service provision should be accommodating. Some studies suggested enhancing primary care as a means of reducing the ED crowding [21, 115], while another study proposed the optimization of translation services to reduce crowding [21]. Others recommended premises related interventions such as expanding or opening an additional ED [116, 117], hallway emergency bed [9] and increasing hospital bed capacity [118] as a solution to prevent crowding.

The emergency healthcare service provision should meet the needs of the patients. Some studies suggested enhancing primary care as a means of reducing ED crowding [21, 115], while another study proposed the optimization of translation services to reduce crowding [21]. Other suggestions were premises related interventions such as expanding or opening additional ED [116, 117], hallway emergency bed [9] and increasing hospital bed capacity [118] as a solution to prevent crowding. Some studies showed reporting of ED utilization to a pediatric specialist was correlated with a cultural and method development to preferentially manage patients with essential matters in the office [119, 120].

### Strengths and limitation

This study attempted to characterize the details of the challenges, emergency patients and emergency clinical staff reactions to clinical emergencies, and the strategies and tactics followed by healthcare service organizations and front-line staff to tackle ED crowding and related issues. Comprehensive keywords and terms to cover all relevant published studies on ED crowding from the PubMed and Embase databases were utilized. The qualities of the studies were evaluated by appropriate checklists and excluded those with low-level quality. However, the systematic review study was limited to studies published only in the English language. The included original studies did not utilize a unique definition for EDs crowding measurement, which led to the inclusion of all ED crowding-related information in the synthesis.

## Conclusions

ED overcrowding is a multi-facet issue which affects by patient-related factors and emergency service delivery. Crowding of the EDs adversely affected individual patients, healthcare delivery systems and communities. The identified issues concern organizational managers, leadership, and operational level actions to reduce crowding and improve emergency healthcare outcomes efficiently. This systematic review study showed the importance of the integrated response to emergencies and emergency related overcrowding and consequences to better address the healthcare needs of emergency patients and effectiveness of healthcare service delivery facilities. Also, multiple health service organization and operational level responses to emergency-related crowding and their consequences were identified.

## Data Availability

The datasets used and/or analyzed during the current study are available from the corresponding author on reasonable request.

## Abbreviations

CASP: Critical Appraisal Skills Programme
ED: Emergency Department
LOS: Length of Stay
LWBS: Left without Being Seen
MeSH: Medical Subject Headings
